# Protocol for a multi-centre, definitive randomised controlled trial of the effectiveness of Individual Placement and Support for employment support among people with alcohol and drug dependence

**DOI:** 10.1186/s13063-020-4099-4

**Published:** 2020-02-11

**Authors:** John Marsden, Paul Anders, Helen Clark, Kyriacos Colocassis, Brian Eastwood, Jonathan Knight, Alexandra Melaugh, David Quinn, Virginia Wright, Jez Stannard

**Affiliations:** 1 Alcohol, Drugs, Tobacco and Justice Division, Health Improvement, Public Health England, Wellington House, 133-155 Waterloo Road, London, SE1 8UG UK; 2 Addictions Department, Division of Academic Psychiatry, Institute of Psychiatry, Psychology and Neuroscience, King’s College London, London, UK

**Keywords:** Individual Placement and Support, Alcohol, Opioids, Drugs, Dependence

## Abstract

**Background:**

Unemployment is highly prevalent in populations with alcohol and drug dependence and the employment support offered in addiction-treatment programmes is ineffective. Individual Placement and Support (IPS) is an evidence-based intervention for competitive employment. IPS has been extensively studied in severe mental illness and physical disabilities, but there have been no formal randomised controlled trials (RCTs) in alcohol and drug dependence. The Individual Placement and Support for Alcohol and Drug Dependence (IPS-AD) study should determine whether IPS for patients with alcohol use disorder (AUD), opioid use disorder (OUD) and other drug use disorder is effective.

**Design/methods:**

The IPS-AD study is a seven-site, pragmatic, two-arm, parallel-group, superiority RCT. IPS-AD includes a realist process evaluation. Eligible patients (adult, unemployed or economically inactive for at least 6 months and wishing to obtain open job market employment and enrolled in ongoing community treatment-as-usual (TAU; the control condition) in England for AUD, OUD and other drug use disorders) will be randomised (1:1) to receive TAU and any standard employment support, or TAU plus IPS (the experimental condition) for 9 months with up to 4 months of in-work support. The primary outcome measure will be competitive employment status (at least 1 day (7 h)) during an 18-month follow-up, determined by patient-level, trial-data-linkage with national tax and state benefit databases. From meta-analysis, an 18% target difference on this measure of vocational effectiveness (for the experimental intervention) and a two-sided 5% level of statistical significance, will require a minimum target sample of 832 participants to achieve 90% power for a pre-registered, mixed-effects, multi-variable logistic regression model. A maximum-likelihood multiple-imputation approach will manage missing outcome data. IPS-AD has six vocational secondary outcome measures during the 18-month follow-up: (1) total time in competitive employment (and corresponding National Insurance contributions and tax paid); (2) time from randomisation to first competitive employment; (3) number of competitive job appointments; (4) job tenure (length of longest held competitive employment); (5) sustained employment (tenure in a single appointment for at least 13 weeks); and (6) job search self-efficacy. A primary cost-benefit analysis and a secondary cost-effectiveness analysis will be done using the primary outcome and secondary vocational outcomes, respectively and will include addiction treatment and social and health outcomes and their associated reference costs. The process evaluation will address IPS implementation and delivery.

**Discussion:**

The IPS-AD study is the first large-scale, multi-site, definitive, superiority RCT of IPS for people with alcohol and drug dependence. Findings from the study will have substantial implications for service delivery.

**Trial registration:**

ISRCTN Registry, ID: ISRCTN24159790. Registered on 1 February 2018.

## Background

Employment is integral to society and a source of economic resource, and an important determinant of personal role identity and life functioning [1]. Job loss and unemployment are associated with stress, poverty, illness and mortality [2–4].

Developed by Becker, Bond and Drake and others at Dartmouth College in the USA, Individual Placement and Support (IPS) is an evidence-based intervention to help people participate in the open competitive labour market [5, 6]. IPS is founded on principles of personal preference, rapid job search, minimal pre-vocational training, and ‘in-work support’ to maintain employment. IPS has been widely studied among people with severe mental illness and physical disability. In these populations, Frederick and VanderWeele reported a meta-analysis of 30 randomised controlled trials (RCTs) of IPS versus standard employment support [7]. In these studies, participants allocated to IPS were more likely to obtain work (relative risk 1.63; 95% Confidence Interval (CI) 1.46–1.82); achieve greater job tenure (defined as duration of longest held competitive employment; Cohen’s *d* 0.55; 95% CI 0.33–0.79); work for longer during follow-up (*d* 0.46; 95% CI 0.35–0.57) and have more income (*d* 0.48; 95% CI 0.36–0.59).

In England in 2017/2018, there was a very high rate of unemployment among people with alcohol and drug dependence (diagnosed as alcohol use disorder (AUD) and opioid, cocaine and other drug use disorder in the *Diagnostic and Statistical Manual of Mental Disorders* (*5th edition*) (*DSM-5*) [8]). Among 75,800 people treated in the community with AUD, 70% were unemployed. Among 141,200 patients with OUD, 85% were unemployed; and among 24,200 patients with another drug use disorder, 68% were unemployed [9]. In contrast with the overall rate of unemployment and economic inactivity in the United Kingdom (UK) in that year – approximately 4% and 21%, respectively^1^ – these are very high rates of unemployment and reflect the ineffectiveness of current employment support provision. In the UK, there are substantial social and economic costs associated with alcohol-related problems (£21.5 billion [10]) and drug-related problems (£20 billion [11]). These include support provided from family and carers; costs associated with the provision of non-elective National Health Service (NHS) hospital treatment (accident and emergency (A&E), outpatient and inpatient care); mortality; loss in productivity; and crime.

Aside from a single, small-scale pilot study of IPS for patients receiving medication treatment for OUD [12], IPS has not been evaluated for people with alcohol and drug dependence. The gap was identified by a UK-government-commissioned independent review [13], and the Work and Health Unit (a collaboration between the Department for Work and Pensions (DWP) and the Department of Health and Social Care (DHSC)) invited researchers from Public Health England (PHE) to submit a proposal for a definitive, multi-centre, RCT of IPS.

This article describes the protocol for the Individual Placement and Support for people with Alcohol and Drug dependence (IPS-AD) trial.

## Methods

### Design

**Fig. 1:**
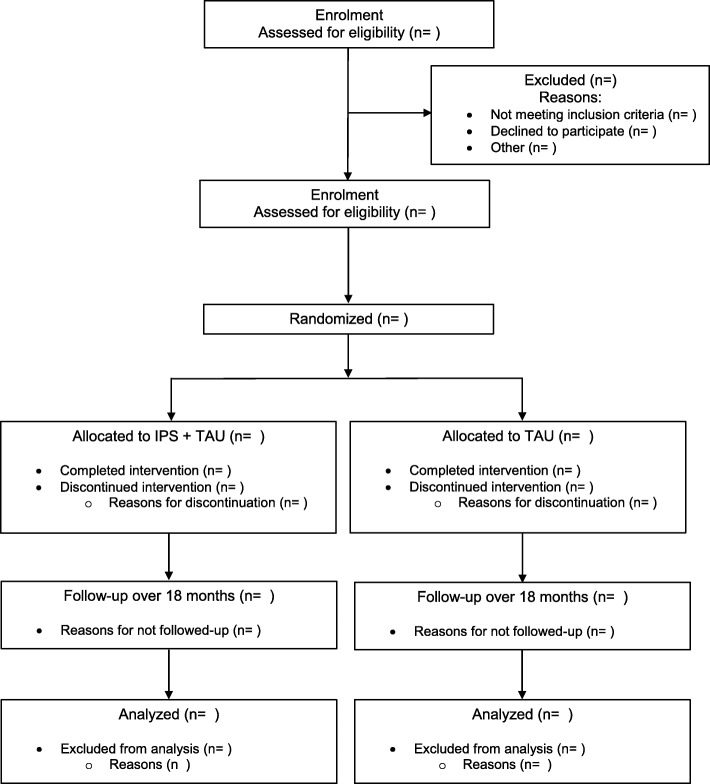
Consolidated Standards of Reporting Trials (CONSORT) flow of participants

IPS-AD is a pragmatic, multi-centre, two-arm, parallel-group, superiority RCT, with a realist process evaluation. The aim of the study is to determine the effectiveness of IPS to help people with AUD, OUD and other drug use disorders to obtain employment in the open competitive job market. These questions will be answered by pre-registered analyses of primary and secondary outcomes recorded across an 18-month follow-up after randomisation (Fig. 1). There are also planned analyses of outcome mediation and effectiveness over the longer term.

After enrolment, all study participants will continue to be enrolled in addiction treatment (co-ordinated by a clinician called a keyworker) and may receive standard employment support (conventionally termed treatment-as-usual (TAU) – the control condition), or they will continue to be enrolled in addiction treatment, and may receive standard employment support, and enrolled in IPS – the intervention condition.

The study will be reported following the CONSORT Guideline (Consolidated Standards of Reporting Trials; http://www.consort-statement.org/) extension for non-medication trials [14] and the Template for Intervention Description and Replication (TIDieR) Checklist for reporting interventions [15]. This protocol has been written following the Standard Protocol Items: Recommendations for Interventional Trials (SPIRIT) Checklist for intervention trials [16] – see Additional file 1: Table S1.

IPS-AD will be conducted following the principles of the Declaration of Helsinki [17], the Medical Research Council Guidelines for Good Clinical Practice [18], and the NHS Research Governance Framework [19]. Participants will have the right to withdraw from the trial at any time without giving a reason. There is no anticipated harm so no compensation arising from trial participation.

### Study population and setting

Study populations will be adults, unemployed and economically inactive^2^ enrolled in community treatment for AUD, OUD or another drug use disorder. The minimum total target sample will be 832 participants. The plan is to include seven clinical recruitment sites in Birmingham, Blackpool, Brighton and Hove, Derbyshire, London Borough of Haringey, Sheffield and Staffordshire. Each site will be a NHS, not-for-profit, or private organisation providing community/outpatient treatment for alcohol and drug dependence. According to the treatment capacity of each site, two or more Employment specialists (ES) will deliver IPS.

### Participant inclusion criteria

Patients will be eligible to take part if they meet the following inclusion criteria:

1. Aged 18–65 years
2. Enrolled in treatment for drug or alcohol use disorder for at least 14 days with current diagnosis of a specified drug and/or alcohol use disorder
3. Unemployed or inactive at study screening visit for at least 6 months with a declared wish to obtain open job market employment
4. Able to attend the community addiction clinic as required in the protocol
5. Able to communicate (verbal and written English) at a level required to engage with a psychosocial intervention
6. Able to provide a personal National Insurance Number (NINO) to facilitate data linkage^3^

### Participant exclusion criteria

Otherwise eligible patients will not be able to join the study if one or more of the following exclusion criteria are met:

1. Currently receiving detoxification treatment for drug or alcohol withdrawal
2. Clinically significant (or otherwise uncontrolled) severe mental health, intellectual disability, organic brain disease or dementia, or physical disability that is judged by the local clinical lead to prevent the person accepting IPS
3. Suicide planning (past month) or suicide attempt (past 6 months)
4. Current legal proceedings which are likely to result in imprisonment
5. Enrolment in an IPS trial, or in the past 6 months
6. Previously enrolled in the IPS-AD study

### Procedure

All keyworkers and ES will complete National Institute for Health Research’s Good Clinical Practice (GCP) training. Potential participants will be referred to an ES in the clinical team to discuss the study. The King’s College London Clinical Trials Unit will programme and independently manage the study randomisation system on a secure website. Immediately after securing informed consent (IPS-AD Participant Information Sheet (PIS) and Participant Consent Form (PCF) is available from the corresponding author on request) and completion of baseline study questionnaires, the ES will access the randomisation website to assign the participant to the intervention or control condition. Group allocation (ratio 1:1) will be done using block randomisation (with varying block size) stratified by site, clinical diagnosis (AUD, OUD, other drug use disorder) and work history (1 month or less versus more than 1 month of paid employment in the last 5 years).

The randomisation system will immediately confirm the participant’s allocation to IPS or TAU by email. The ES will then inform the participant and their keyworker. Participants assigned to TAU will be given an information pack containing details of standard employment support services available locally and will have no further contact with the ES. It will not be feasible to blind clinicians to trial condition allocation. Enrolment in TAU will be ongoing after the trial according to patient preference and local policy.

### IPS intervention principles and delivery

IPS will be offered as an individual (one-to-one) intervention for 9 months with up to four additional months of in-work support if competitive employment is attained. IPS will be provided without restriction due to job readiness, work history, qualifications and homelessness status. In weekly sessions in the first month, the ES will: (1) discuss opportunities to work and will give advice on welfare benefits, including the availability of in-work benefits; (2) develop a vocational profile of the participant’s skills, experience and employment preferences; (3) help the participant to write or update their curriculum vitae/resumé; and (4) help the participant to implement a rapid job search. As appropriate, the ES will contact local employers and help the participant to complete job applications and prepare for interviews. The ES will seek to develop relationships with local employers and discuss opportunities to tailor work for people recovering from alcohol and drug dependence.

After the first month, IPS sessions will be scheduled approximately fortnightly, with additional phone and email contact provided as needed. Once the participant starts work, the ES will offer in-work support. This support will be approximately weekly contacts in the first month, then fortnightly. The ES will discuss how the participant is adapting to their new job; assist with any referral for medical treatment; and, with consent, seek to discuss job-flexibility issues with the employer (e.g. adjusting shift patterns to enable the participant to collect treatment medication from a retail pharmacy).

### Standard employment support

Vocationally, participants randomised to the TAU control group will receive standard employment support only. For participants receiving Jobseeker’s Allowance (JSA) or Universal Credit (UC) with all work-related requirements, TAU will usually involve support from Jobcentre Plus (JCP) and/or the Work and Health Programme (WHP). TAU may also include standard employment support provided by the addiction-treatment programme or other local services.^4^

### IPS training for ES

Training for ES will consist of a continuing professional development (CPD) accredited 2-day course facilitated by the Centre for Mental Health (CMH) and a 12-week online ES practitioner skills course provided by the USA IPS Employment Center. IPS-AD recruitment sites will be encouraged to look for CPD opportunities for the ES team, including forging links with the local IPS Centre of Excellence and with IPS Grow (the latter an IPS capacity building network in the NHS and mental health services with support funding from NHS England and the DWP).

### IPS fidelity

To help develop and maintain IPS fidelity, IPS-AD will use the 25-item Individual Placement and Support Fidelity Scale (IPS-25) [20]. The IPS-25 was adapted to the UK context by the CMH [21] and has three sections: staffing, organisation and services. Independent reviewers score items on the scale using a 5-point response format (1 = no implementation; 5 = full implementation), with intermediate numbers representing progressively greater degrees of implementation. The maximum score is 125 points. During the study, the CMH will co-ordinate all fidelity reviewers with another organisation called Social Finance (SF). CMH and SF raters will complete the IPS-25 with access to multiple sources of information, including interviews with study participants, ES and managers, as well as reviewing case records and observing the day-to-day practice of the IPS and clinical teams. From the start of the study, there will be planned fidelity reviews at each site at 5–7 months and 15–18 months. CMH and SF will provide each site with a detailed report of their IPS-25 score and will provide advice on how this can be improved.

### Realist process evaluation

RAND Europe and the CMH will conduct the IPS-AD process evaluation. Theory-driven and following realist principles, this will investigate IPS and clinical practice and answer questions of the characteristics of patients and IPS exposure that are associated with competitive employment outcomes. An initial focus will be on any obstacles encountered during the set-up of IPS in each site and how IPS is integrated into routine procedures. In each site, RAND Europe and CMH researchers will conduct personal interviews (each audio-recorded with permission) with a random sample of participants allocated to IPS and TAU as well as a convenience sample of ES, treatment service commissioners, managers, keyworkers, local JCP staff and employers.

### Primary outcome measure

The primary outcome of the study is competitive employment status. This outcome will be met if the participant obtains at least 7 h (i.e. 1 day) of employment in the open competitive job market at any time following randomisation to the end of an 18-month follow-up. This outcome measure has been commonly used in IPS efficacy studies and meta-analysis. We will determine this outcome by linking participant-level information with the Department for Work and Pensions’ (DWP) Work and Pensions Longitudinal Study (WPLS) database.

### Secondary outcome measures

#### Vocational

IPS-AD includes the following secondary vocational outcome measures:

- Total time (days) in competitive employment (and corresponding National Insurance contributions (NIC) and tax paid)
- Time (days) from randomisation to first competitive employment
- Number of competitive job appointments
- Job tenure (length of longest held competitive employment)
- Sustained employment (tenure in a single appointment for at least 13 weeks)^5^
- Job search self-efficacy

The time and count-based vocational outcomes will be determined from extracts of the WPLS database using the dates of starting and stopping competitive employment. NIC and tax records from randomisation to the end of follow-up will be determined using HMRC’s Real Time Information system (RTI).

Job search self-efficacy (for a mediation analysis of outcome) will be assessed by the six-item Job Search Self-Efficacy: Behaviour scale (JSSE-B) [22]. The JSSE-B includes measures of confidence in making a good impression, making a good application, and using friends and contacts to discover vacancies. The JSSE-B has been shown to predict job search behaviour. Site clinicians will complete the JSSE-B during personal interviews at baseline, 6 months, 12 months and 18 months or at treatment exit if earlier (the latter if feasible).

#### Alcohol and drug dependence treatment-related

For the secondary outcomes and economic analyses, the following alcohol and drug dependence treatment-related outcomes will be included:

- Alcohol consumption, drug use, drug injecting
- DSM-5 AUD, OUD and other drug use disorder remission status
- Total time enrolled in alcohol and drug use disorder treatment
- Number of AUD, OUD and other drug use disorder treatment episodes
- Treatment status at end of follow-up (enrolled, left successfully, left unsuccessfully, deceased)

Alcohol and drug use (recall: past 28 days) will be self-reported to site clinicians using the Treatment Outcomes Profile (TOP) [23]. The TOP is the English national outcomes monitoring instrument for publicly funded treatment services for drug and alcohol dependence. TOP data is uploaded to the National Drug Treatment Monitoring System (NDTMS). The TOP uses a structured, calendar-prompt, ‘timeline follow-back’ procedure [24] to maximise accuracy of drug and alcohol use reporting. The TOP will be administered by site clinicians as a structured personal interview at baseline, 6 months, 12 months,18 months and exit.

DSM-5 remission status will be assessed by personal interview by site clinicians at baseline, 6 months, 12 months and 18 months using the Structured Clinical Interview for DSM-5 (clinician version; SCID-5-CV) [25]. This has a checklist of 11 symptoms (each coded ‘present’ or ‘absent’) to diagnose disorder presence and severity (no symptoms = disorder not present; 1–3 symptoms = mild; 4–5 symptoms = moderate; ≥ 6 symptoms = severe). We will use the American Psychiatric Association’s definition for AUD and OUD remission (i.e. zero criteria except craving and using the ‘on maintenance therapy’ specifier as appropriate and not including tolerance and withdrawal item if the patient is adherent to their prescription).

NDTMS records will be used to determine each participant’s total time in treatment in the community and in prison, as well as the number of treatment episodes and status at the end of follow-up. For data modelling, we will record TOP and treatment exposure data for 18 months prior to randomisation for those members of the cohort with a history of prior treatment for alcohol and drug dependence.

#### Social and health

For the economic analysis, the following social and economic outcomes will be included:

- Welfare payments (i.e. Job Seeker’s Allowance (JSA); Income Support (IS) Employment and Support Allowance (ESA); Universal Credit (UC); Housing Benefit (HB) and Tax Credits (TC))
- NHS hospital attendances and admissions (A&E, outpatient and inpatient)
- EuroQol 5-dimension, 5-level health survey (EQ-5D-5 L)
- Mortality
- Convictions
- Prison sentences

JSA, IS, ESA, UC and HB welfare payments will be determined from the dates of starting and stopping receipt of each benefit type, or changes to levels of payment of each type, recorded in the following DWP databases: National Benefits Database (JSA, IS and ESA); and Universal Credit Official Statistics (UC), Housing Benefit Extract (HB). Tax Credit spells and amounts will be determined by use of HMRC’s General Matching System (GMS).

Hospital care will be recorded by Hospital Episode Statistics (HES; PHE is the data controller). The Office for National Statistics’ (ONS) register of births and deaths will record mortality.

Convictions and prison sentences will be recorded from extracts from the Police National Computer (PNC) and the National Offender Management Information System (p-NOMIS), respectively (both operated by the Ministry of Justice).

The EQ-5D-5 L [26] is a brief generic scale recording mobility, self-care, usual activities, pain/discomfort, anxiety/depression, with a 0 to 100-point vertical visual analogue scale (VAS) measuring overall health status. Site clinicians will complete this measure during personal interviews at baseline, 6 months, 12 months and 18 months and exit. HES, DWP and HMRC welfare payments, PNC and p-NOMIS data will be recorded from randomisation to the end of follow-up. For data modelling, we will also record these outcomes for 18 months prior to randomisation.

The schedule of enrolment, allocation, interventions and assessments is summarised in Table 1.

**Table 1:**
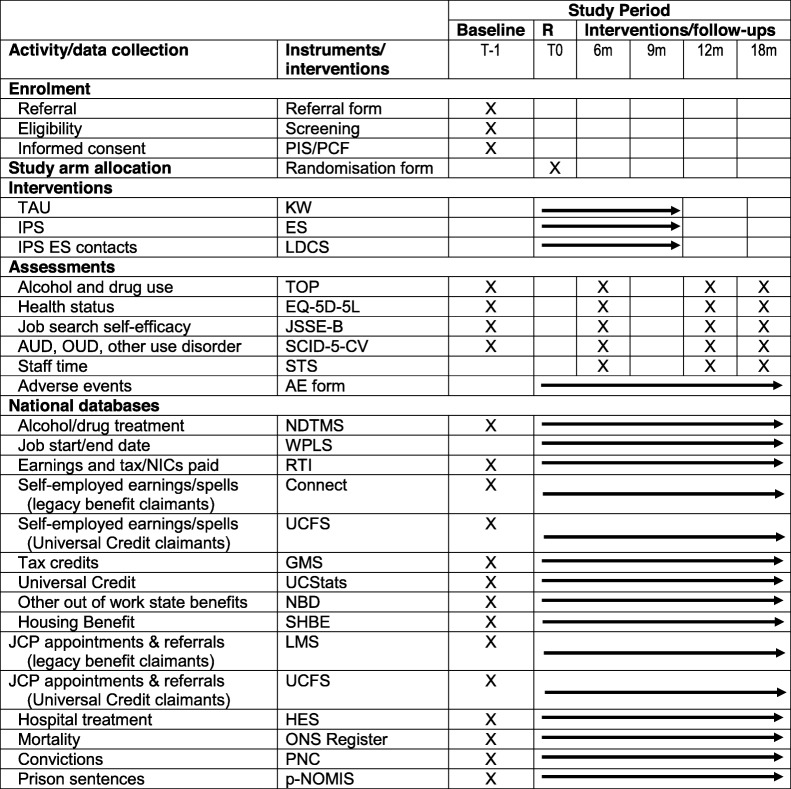
Schedule of enrolment, allocation, interventions and assessments

*AE form* adverse events form, *AUD* alcohol use disorder, *EQ-5D-5 L* EuroQol five-level health status, *ES* employment specialist, *FSS* Universal Credit Full Service System (Department for Work and Pensions), *GMS* General Matching System (Her Majesty’s Revenue and Customs), *HES* Hospital Episode Statistics (Public Health England), *IPS* Individual Placement and Support (the experimental intervention), *JSSE-B* Job Search Self-efficacy scale, *KW* treatment service keyworker, *LMS* Labour Market System, Department for Work and Pensions, *LDCS* local data collection system (Public Health England), *LMS* Labour Market System (Department for Work and Pensions), *NDTMS* National Drug (and alcohol) Treatment Monitoring System, *ONS* Register deaths registered in England and Wales (Office for National Statistics), *OUD* opioid use disorder, *PIS/PCF* Participant Information Sheet/Participant Consent Form, *PNC* Police National Computer (Ministry of Justice), *p-NOMIS* National Offender Management Information System (Ministry of Justice), *R* randomisation, *RTI* Real Time Information database (Her Majesty’s Revenue and Customs), *SCID-5-CV* structured clinical interview for DSM-5 (alcohol and drug use) disorders, clinician version, *SHBE* Housing Benefit extract (Department for Work and Pensions), *STS* Staff Time Survey, *TAU* treatment as usual, *TOP* Treatment Outcomes Profile, *UCStats* Universal Credit Official Statistics (Department for Work and Pensions), *WPLS* Work and Pensions Longitudinal Study (Department for Work and Pensions), *UCFS* Universal Credit Full Service System Strategic Extract, Department for Work and Pensions, *UCStats* Universal Credit Official Statistics. All database names and acronyms are correct at the time of writing but may be subject to change and this will be noted in the analysis plans

### Sample size

Following preliminary planning, we followed the DELTA^2^ guideline to estimate the minimum sample size for the study [27]. Frederick and VanderWeele’s meta-analysis [7] was used to specify the important and realistic target difference for the primary outcome, with an estimate of its uncertainty. A sensitivity calculation was included if assumptions are mis-specified.

For comparison to the duration of the IPS intervention in IPS-AD, we used the rate of competitive employment status reported by seven superiority trials which evaluated 12 months of IPS (928 participants; outcome rate 0.36 for IPS and 0.13 for TAU; odds ratio (OR) 3.76; 95% CI 2.70 to 5.24). Conservatively, we used the lower bound of the CI (i.e. 2.70; equivalent to an outcome rate of IPS 0.36 and TAU 0.18).

To achieve 90% power to detect this 18% target difference (with a two-sided 5% level of statistical significance and a 20% increase to compensate for missing or inaccurate NINO information) we estimate that 302 participants will be required with AUD and OUD (giving an expected 95% CI estimate for the OR effect within a range from 1.50 to 4.36). If the observed effect falls short of the target difference, we will be able to detect a 15% difference (which we judge is still important) with 83% power (OR 95%, CI 1.24–3.46).

Given the lower number of people in treatment with other drug use disorders, we expect to recruit fewer participants in this group, so it will be realistic to power the analysis at 80%. For the 18% target difference (two-sided, 5% level of significance and with 20% increase for attrition), 228 participants will be needed (95% CI 1.35–4.57).

For the secondary outcome of total time worked, four of the seven trials used for the power calculation for the primary outcome measure in the meta-analysis reported the total number of hours worked (i.e. the sum of all time in all competitive employment during the trial). These four trials recruited 376 participants and the pooled mean difference was 505 h (Hedges’ *g* effect size = 0.54; 95% CI 0.33–0.74). Using this effect size as the realistic mean target difference, the analysis of the secondary outcome for length of competitive employment will have 99% to detect this target difference for the AUD and OUD groups. For the other drug use disorder group, we will be able to detect an effect size ranging from 0.54 to 0.38 with a minimum of 82% power.

With these conservative planning assumptions, a total of 832 participants will be the minimum number of participants required. Mediation analysis and longer-term follow-ups will benefit from a greater sample size, so recruitment is expected to extend well beyond this minimum.

### Study governance

Following signed terms of reference and charter respectively, an independently chaired Trial Steering Committee (TSC) and Data Monitoring Committee (DMC) will oversee study integrity, recruitment, research measure completion and analysis. These committees will include members with addiction service delivery, commissioning or IPS expertise, and patient and public involvement (PPI). The Trial Management Group will be responsible for day-to-day running of the study and members will attend meetings of the oversight committees. After approving the protocol, the TSC and DMC will meet approximately three times each year.

All serious adverse events will be promptly reported to the DMC (for the TSC) and the study sponsor. The chief investigator will have overall responsibility for the trial dataset, supported by the oversight committees. The study may be prematurely discontinued by the sponsor, or for reasons reported by the chair of the DMC to the chair of the TSC.

### Information governance and data linkage

Physical case report forms will be securely stored at each site. Sites will report research data and management information securely via NDTMS. A data submission portal will transfer monthly patient information to the study. Clinical site personnel will use a two-factor authentication before access to submit data.

A bespoke Local Data Collection System (LDCS) will collect data on participant identification characteristics, scheduled and attended IPS support sessions, and self-report job data to facilitate study monitoring. LDCS will be used by the principal investigator (PI) and ES at each site with data sent to the study via secure file transfer. All study data will be stored in password-protected folders within a restricted area of PHE’s network, accessible only by a limited number of authorised analysts. Additionally, there will be physical and other data-security safeguards to protect the data, and audit processes.

The planned deterministic data-linkage procedure (to be described in detail in the pre-registered analysis plan) will be based on the participant’s NINO for vocational outcomes, and NHS number for health-related outcomes. If the event of missing data, linkage will be done utilising the participant’s full first name and surname, date of birth, gender and full or partial postcode or upper-tier local authority of residence. The HES patient identifier will be used to verify that a participant has been linked to a single HES patient. Linkage with offending databases (PNC and p-NOMIS) will utilise a participant’s full first name and surname, date of birth, gender and upper-tier local authority of residence. Data to enable linkage will be transferred from PHE to government departments via a strong password-protected, encrypted, file-transfer protocol. This transfer and linkage protocol will be reviewed periodically and may be enhanced.

### Analysis

Descriptive analysis will summarise the sample in terms of demographic, clinical and vocational history characteristics. For those attaining competitive employment during the study, we will summarise their earnings and income using HMRC’s RTI system.

#### Primary vocational effectiveness

The statistical analysis plan (SAP) will be approved by the trial committees and will be published on the Open Science Framework (OSF; www.osf.io) before it is implemented after data-lock. There will be no interim analyses and specified trial-stopping rules. The analysis of the primary outcome (completed in STATA or R; final code to be placed on OSF) will follow the intention-to-treat (ITT) principle and include all patients in the group to which they are allocated. Alpha will be set at 5% for the primary and secondary outcomes (with associated 95% CIs). The distributions of scale and count measures may be non-normal (skewed), so that test statistics and effect sizes may be computed following appropriate transformation (e.g. natural log to obtain a geometric mean).

Data from all sites will be pooled and the superiority effectiveness estimate for the IPS intervention (adjusted OR and CI) will be determined using a mixed-effects, multi-variable logistic regression model. The model will include the stratification variables and a random intercept for each site to account for clustering. A maximum-likelihood multiple-imputation approach will be used for the management of missing data with a sensitivity comparison to the complete case dataset.

#### Analysis of secondary vocational and alcohol and drug-treatment-related outcomes

The ITT analysis of the secondary vocational and clinical outcomes will be done using appropriate mixed-effects regression models according to each measure (i.e. linear for time-based (total time in employment and treatment); Poisson for count-based (number of appointments; days of alcohol and drug use; number of treatment episodes); logistic for binary outcomes (sustained employment; *DSM-5 remission*); ordinal for treatment exit status; and proportional-hazards (for time to first employment) with measure-appropriate covariates. These models will include site and employment history stratification factors and may include other background variables. Exploratory models will also be separately done for AUD, OUD and other drug use disorder groups. A causal mediation framework analysis will be used to determine the evidence for a theoretical mechanism of change for the IPS intervention using the JSSE-B as a mediator of competitive employment.

After completing the analysis and reporting of the primary and secondary analyses, we plan to undertake exploratory longer-term analyses using the national registry data at 3 years and 6 years – subject to approval for a protocol amendment.

#### Economic analysis

The health economic analysis plan (HEAP) will be approved by the trial committees and will be published on the OSF before data-lock. Using all primary and secondary vocational, treatment-related, social and health outcomes, the analysis will determine whether IPS has a positive net benefit and is cost-effective compared to TAU. Using a cost-benefit ratio, a primary social cost-benefit analysis (CBA) will estimate the extent of additional monetised benefits accrued by the public and the Exchequer from investing in IPS. Costs and benefits will be analysed at the patient level, before and after exposure to IPS and TAU.

Taking an NHS and patient perspective, a secondary cost-effectiveness analysis (CEA) will compare outcomes at baseline and 18 months after trial enrolment to calculate the additional cost per quality-adjusted life-year (QALY), using mortality data and utilities estimated using the EQ-5D-5 L. An incremental cost-effectiveness ratio (ICER) will be estimated to determine whether IPS is cost-effective from the perspective of the health and social care sectors.

Outcomes from national registries will be used to estimate net tax revenue benefits accrued to the Exchequer, along with wider societal and economic benefits, and QALY gains. Official government fiscal, economic and social monetary values will be applied to the difference in events observed pre and post enrolment between the control and the intervention arms. For example, hospital activity will be valued by attaching average unit costs per episode derived from the Personal Social Services Research Unit (PSSRU) [28] or the national reference sheet [29] and criminal activity will be valued using the Home Office social and economic costs of crime [30].

The unit costs of IPS at each site will be estimated from information from the provider on site delivery using a Staff Time Survey (STS) of direct and indirect time spent on delivering IPS and delivering research. This will be conducted on three occasions during the study (i.e. at 6 months, 12 months and 18 months) to remove noise from the data collection exercise. The unit costs of TAU (including JCP appointments) will be calculated for both the control and the intervention arms of the trial using the Universal Credit Full Service (UCFS) strategic extract for Universal Credit claimants and the DWP Labour Market System (LMS) for legacy benefit claimants. We will work towards incorporating the costs of referrals to other labour market programmes, such as the WHP, using the same data sources.

All costs will be multiplied by the market forces factor (MFF) developed by the NHS to adjust for the unavoidable geographical cost differences by site and differential labour and building costs.

The within-trial primary and secondary analysis will be conducted after all participants have been followed-up for 18 months after trial enrolment. After completing the analysis and reporting of the within-trial primary and secondary economic analyses and sensitivity checks as specified in the HEAP, we envisage undertaking an exploratory longer-term economic analysis phase of the national registry data at 3 years and 6 years, subject to approval for a protocol amendment.

## Discussion

There is a complex and costly relationship between unemployment and alcohol and drug use and dependence. Harmful drinking is a risk factor for job loss [31] and, in some groups, unemployment predicts higher levels of drinking [32]. During economic recession, there is evidence that some people start to drink more and harmfully after losing their job [33]. Opioid use disorder (OUD) is associated with chronic unemployment [34, 35]. Untreated, OUD is associated with a reduced likelihood of job-seeking activity and finding work [36, 37].

IPS is a promising candidate intervention for people with alcohol and drug dependence who are seeking work; but there have been no formal trials. IPS-AD will provide policy-makers and treatment service commissioners with a definitive estimate of IPS effectiveness.

There will be several challenges to undertaking a pragmatic effectiveness RCT in community treatment services operated by the NHS, non-governmental and commercial providers. While ES are funded posts for the study, the keyworkers will be dividing their time between their primary clinical role and research tasks (e.g. completion of research measures). It may be challenging to secure the same rate of research follow-up between the two arms of the study. However, this will not affect the primary outcome because of the data-linkage design.

A strength of the study will be the causal mediation analysis and process evaluation to investigate IPS change mechanisms. For the former, the job-search self-efficacy concept (here measured by the JSSE-B instrument) has been frequently used in IPS research. One acknowledged limitation of the study is that we may not be able to determine competitive employment status for some participants who pursue self-employment due to the timeline for submitting self-assessment tax returns to HMRC. The current system in the UK is for a paper tax return to be submitted within 6 months after the end of a tax year and 9 months if the tax return is online. With the IPS-AD 18-month follow-up completed at the end of March 2021, determination of competitive employment would not be known for at least 10 months after this point (i.e. January 2022). The CBA and CEA questions for the sub-population of participants who register for self-employment and attain at least 7 h of paid work will, therefore, be addressed in a longer-term follow-up after the analysis of the primary outcome has been reported. Self-employment start, and end dates and associated earnings will be monitored using HMRC Connect and the UCFS databases where available.

In the UK, there is a high prevalence of unemployment among populations enrolled in treatment for alcohol and drug dependence, and a pressing need for effective employment interventions. If IPS is to prove effective, there will be NHS health savings, along with crime and employment benefits annually for the economy and the Exchequer [38]. We anticipate that the IPS-AD trial will make a substantial contribution to policy and practice.

## Trial status

IPS-AD was registered on ISRCTN (ID: ISRCTN24159790) on 1 February 2018. This article refers to version 1.0 of the approved protocol (23 May 2018). The first participant was enrolled in the study on 8 May 2018. The trial is ongoing and recruiting participants. The last day of participant recruitment will be 30 September 2019. The ISRCTN entry was edited on 20 June 2019 to show that participant recruitment is to be extended to 30 September 2019.

## Supplementary information

**Additional file 1: Table S1.** Standard Protocol Items for Randomised Trials (SPIRIT) 2013 Checklist.

## Data Availability

A trial implementation guide developed by the study team is available from the corresponding author on request. This manuscript does not contain any data. Results from the trial will be submitted to academic journals and via press release and related communications via the sponsor.

## Abbreviations

A&E: Accident and emergency
AE: Adverse event
AUD: Alcohol use disorder
CBA: Cost-benefit analysis
CEA: Cost-effectiveness analysis
CI: Confidence Interval
CMH: Centre for Mental Health
Connect: HMRC database
CONSORT: Consolidated Standards of Reporting Trials
CPD: Continuing professional development
DHSC: Department of Health and Social Care
DMC: Data Monitoring Committee
DSM-5: *Diagnostic and Statistical Manual of Mental Disorders (5th edition)*
DWP: Department for Work and Pensions
EQ-5D-5 L: EuroQol 5-dimension, 5-level version
ES: Employment specialist
ESA: Employment and Support Allowance
FSS: Full Service System
GCP: Good Clinical Practice
GDPR: General Data Protection Regulation
GMS: General Matching System
HB: Housing benefit
HEAP: Health economic analysis plan
HES: Hospital Episode Statistics
HMRC: HM Revenue and Customs
ICER: Incremental cost-effectiveness ratio
IPS: Individual Placement and Support
IPS-AD: Individual Placement and Support for people with Alcohol and Drug dependence trial
IRAS: Integrated Research Application System
ITT: Intention-to-treat
JSA: Jobseeker’s Allowance
JSSE-B: Job Search Self-Efficacy: Behaviour scale
LDCS: Local Data Collection System
LMS: Labour Market System, DWP management information system
MFF: Market forces factor
MOJ: Ministry of Justice
NBD: National Benefits Database
NDTMS: National Drug Treatment Monitoring System
NHS: National Health Service
NIC: National Insurance contributions
NIHR: National Institute for Health Research
NINO: National Insurance Number
ONS: Office for National Statistics
OR: Odds ratio
OSF: Open Science Framework
OUD: Opioid use disorder
PCF: Participant Consent Form
PHE: Public Health England
PI: Principal investigator
PIS: Participant Information Sheet
PNC: Police National Computer
p-NOMIS: Prison National Offender Management Information System
PPI: Patient and public involvement
QALY: Quality-adjusted life-year
RCT: Randomised controlled trial
REGG: Research Ethics and Governance Group
RTI: HMRC Real Time Information system
SAP: Statistical analysis plan
SCID-5-CV: Structured Clinical Interview for DSM-5 Disorders – Clinician Version
SF: Social finance
SHBE: DWP Housing Benefit database
SPIRIT: Standard Protocol Items: Recommendations for Interventional Trials
STS: Staff Time Survey
TAU: Treatment as usual (standard employment support)
TIDierR: Template for Intervention Description and Replication
TOP: Treatment Outcomes Profile
TSC: Trial Steering Committee
UC: Universal Credit
UCFS: Universal Credit Full Service Strategic Extract
UCStats: Universal Credit Official Statistics
VAS: Visual analogue scale
WPLS: Work and Pensions Longitudinal Study, DWP database

## Footnotes

1 Derived from the Labour Force Survey, data on unemployment and economic inactivity are published by the UK Office for National Statistics (ONS) quarterly.

2 ONS defines ‘economically inactive’ as a person aged 16 years and over who is not employed, has not sought work in the last 4 weeks and is not available to start work in the next 2 weeks.

3 In the UK, the NINO is a unique nine-item alpha-numeric code issued to each resident and used by DWP and HMRC databases

4 For many participants in receipt of health-related unemployment benefits, there may be little or no contact with public employment services, although they may still be able to access employment support through the treatment partnership.

5 This outcome has been used to indicate job sustainment in other IPS studies in the UK as well as several UK labour market programmes (see: https://www.gov.uk/government/publications/work-programme-dwp-provider-guidance).
